# Construction of a Necroptosis-Related miRNA Signature for Predicting the Prognosis of Patients With Hepatocellular Carcinoma

**DOI:** 10.3389/fgene.2022.825261

**Published:** 2022-04-12

**Authors:** Tongyu Meng, Qingfeng Wang, Yufeng Yang, Yanling Ren, Yan Shi

**Affiliations:** Liaoning University of Traditional Chinese Medicine, Shenyang, China

**Keywords:** hepatocellular carcinoma, miRNAs, signature, necroptosis, prognosis

## Abstract

**Background:** Many miRNAs have been demonstrated to be associated with the prognosis of hepatocellular carcinoma (HCC). However, how to combine necroptosis-related miRNAs to achieve the best predictive effect in estimating HCC patient survival has not been explored.

**Methods:** The mRNA and miRNA expression profile were downloaded from a public database (TCGA-LIHC cohort). Necroptosis-related genes were obtained from previous references, and necroptosis-related miRNAs were identified using Pearson analysis. Subsequently, differential expression miRNAs (DEms) were identified in HCC and paracancer normal samples based on necroptosis-related miRNA expression. The whole set with HCC was randomized into a training set and testing set (1:1). LASSO-Cox regression analysis was used to construct an miRNA signature. Multiple statistical methods were used to validate the clinical benefit of signature in HCC patients, including receiver operator characteristic (ROC) curves, Kaplan–Meier survival analyses, and decision curve analysis (DCA). The downstream target genes of miRNAs were obtained from different online tools, and the potential pathways involved in miRNAs were explored. Finally, we conducted RT-qPCR in SK-HEP-1, THLE-3, and HUH-7 cell lines for miRNAs involved in the signature.

**Results:** The results showed that a total of eight specific necroptosis-related miRNAs were screened between HCC and adjacent tissues in the training set. Subsequently, based on the aforementioned miRNAs, 5-miRNA signature (miR-139-5p, hsa-miR-326, miR-10b-5p, miR-500a-3p, and miR-592) was generated by LASSO-Cox regression analysis. Multivariate Cox regression analysis showed that the risk scores were independent prognostic indicators in each set. The area under curves (AUCs) of 1 year, 3 years, 5 years, and 7 years were high in each set (AUC >0.7). DCA analysis also revealed that the risk score had a potential benefit than other clinical characteristics. Meanwhile, survival analysis showed that the high-risk group showed low survival probabilities. Moreover, the results of enrichment analysis showed that specific miRNAs were mainly enriched in the cAMP signaling pathway and TNF signaling pathway. Finally, the results of RT-qPCR were consistent with the prediction results in public databases.

**Conclusion:** Our study establishes a robust tool based on 5-necroptosis-related miRNAs for the prognostic management of HCC patients.

## Introduction

By 2025, more than 1 million new cases of hepatocellular carcinoma (HCC) will be diagnosed each year worldwide, a serious situation that will pose a major challenge to global healthcare; more importantly, the 5-year survival rate for HCC patients has decreased by 20% globally and as low as 12% in Asian countries such as Japan. (Llovet et al., 2021). Smoking, drinking, and viral infection are risk factors for the occurrence and prognosis of HCC (El-Serag, 2011). In addition, local recurrence and metastasis reduce the survival rate in HCC patients (Di Sandro et al., 2019). Therefore, intricate etiological factors and heterogeneity of HCC make prognostic prediction challenging. There is an urgent need to develop a new prognostic model considering the limitations of HCC treatment strategies.

Necroptosis is a cell death independent of caspase (Linkermann and Green, 2014) including the following characteristics: incomplete cell membrane, intracellular metabolic abnormalities, and release of inflammatory factors. (Christofferson and Yuan, 2010). It is worth noting that necroptosis plays an important role in the occurrence and development of various diseases such as neurodegenerative diseases (Dionísio et al., 2020), ischemic cardiovascular (Ruan et al., 2019), and cancers (Gong et al., 2019). Interestingly, necroptosis has been shown to play a dual role in cancer. On the one hand, the hub regulators of necroptosis can promote metastasis and progression of cancer (Strilic et al., 2016); on the other hand, necroptosis can also prevent tumor development when apoptosis function in cancer cells is impaired (Long and Ryan, 2012). Meanwhile, necroptosis is regulated by intracellular signaling factors such as tumor necrosis receptor factor (TNFR) (Lalaoui et al., 2015), pattern recognition receptors (PRRs) (Li et al., 2012), and T-cell receptors (TCRs) (Hitomi et al., 2008). At present, relevant reports have revealed the regulation mechanism of necroptosis in HC. Xiang et al. (2021) discovered how CX32 induces necroptosis, and high CX32 expression may be represented as a resistant role to apoptosis inducers. Meanwhile, mosaic mouse models were used to reveal how necroptosis microenvironment directs lineage commitment in liver cancer (Seehawer et al., 2018). In addition, it is reported that some compounds can induce necroptosis to treat cancer. These evidence remind us that necroptosis has a potential application value in the treatment of HCC. In addition, microRNAs (miRNAs) are small molecules encoded by the genomes of eukaryotes, similar to siRNA (Bartel, 2004). There have been many studies on miRNA in HCC, such as the one where Wang et al. (2017a) found that miR-218 can suppress the metastasis and EMT of HCC cells *via* targeting SERBP1. Moreover, different noncoding RNAs also interact with each other in the progression of HCC. Ding et al. (2020) revealed that hsa_circ_0001955 enhances proliferation, migration, and invasion of HCC cells through miR-145-5p/NRAS axis. Surprisingly, a combination of miRNA signatures in predicting HCC survival was elucidated by Li et al. (2020). In addition, enhancers can act as tissue-specific cis-regulatory elements to positively regulate gene expression by miRNAs (Zhang et al., 2020).

However, no study has systematically used necroptosis-related miRNA to predict the prognosis of HC patients.

In order to solve the aforementioned problem, we downloaded expression data and clinical features of TCGA-LIHC cohort from public database and extracted miRNA data related to necroptosis. Then, a 5-miRNA prognosis signature was constructed by LASOO-Cox regression analysis, and its prognostic ability was verified in different cohorts. The functional enrichment analysis of downstream genes of miRNAs is performed to explore the potential mechanism. Finally, we conducted RT-qPCR in SK-HEP-1, THLE-3, and HUH-7 cell lines for 5-miRNAs involved in the signature.

## Materials and Methods

### Datasets and Pre-Processing

We downloaded the clinical features and miRNA expression of HC patients from The Cancer Genome Atlas (TCGA) database (Wang et al., 2016) *via* the R package “TCGAbiolinks”. The miRNA expression profile in TCGA-LIHC includes 50 normal samples and 375 tumor samples (Table 1). Based on previous studies (Liu et al., 2021a; Visalli et al., 2018), we obtained 16 miRNAs associated with necroptosis, which were miR-495, miR-331-3p, miR-15a, miR-148a-3p, miR-7-5p, miR-141-3p, miR-425-5p, miR-200a-5p, miR-210, miR-223-3p, miR-500a-3p, miR-181-5p, miR-16-5p, hsa-miR-371-5p, hsa-miR-373, and hsa-miR-543 in detail. In addition, 67 necroptosis-related genes were extracted based on previous bioinformatics research (Hu et al., 2022; Huang et al., 2021). We performed Pearson correlation analysis (|cor|>0.15, *p* < 0.05) on 2435 miRNAs and 67 necroptosis-related genes from the raw data [RNA-seq (log FPKM+1 format), miRNA-seq (log RPM +1 format)]. Ultimately, we annotated 144 necroptosis-related miRNAs in TCGA-LIHC from the references and Pearson correlation analysis.

**TABLE 1:** Clinicopathological features of TCGA-LIHC.

Variables	Count	Percentage (%)
Age (mean ± SD)	59.57 ± 13.33	
Status		
Alive	249	66.05
Dead	128	33.95
Gender		
Male	255	67.64
Female	122	32.36
Pathological stage		
Stage I	175	46.42
Stage II	87	23.08
Stage III	3	0.80
Stage IIIA	65	17.24
Stage IIIB	9	2.39
Stage IIIC	9	2.39
Stage IV	2	0.53
Stage IVA	1	0.27
Stage IVB	2	0.53
Unknown	24	6.37
T staging		
T1	185	49.07
T2	93	24.67
T2a	1	0.27
T2b	1	0.27
T3	45	11.94
T3a	29	7.69
T3b	7	1.86
T4	13	3.45
TX	1	0.27
Unknown	2	0.53
N staging		
N0	257	68.17
N1	4	1.06
NX	115	30.50
Unknown	1	0.27
M staging		
M0	272	72.15
M1	4	1.06
MX	101	26.79
Grade		
G1	55	14.59
G2	180	47.75
G3	124	32.89
G4	13	3.45
Unknown	5	1.33

### Calculation of the Risk Score *via* the Necroptosis-Related MicroRNA Signature

The differentially expressed miRNAs (DEms) in normal samples and tumor samples were screened by using “limma” package (Ritchie et al., 2015) in R software. The thresholds in ‘limma’ package were set to adjusted *p*-value < 0.05, and |logFCfilter| = 1. At a ratio of 1:1, the whole set was divided into two using the ‘caret’ package in R software. We performed LASSO-Cox regression analysis in the training set (*p* < 0.05), and the risk score was calculated as follows:

$$\mathrm{risk score}=\overset{n}{\underset{i=1}{\sum }}\mathrm{Coe}f_{i}*x_{i},$$

where Coef is the coefficient, and x is the expression value of each selected miRNA. LASSO is a popular algorithm which was extensively utilized in medical studies (Liu et al., 2022a), (Liu et al., 2022b), (Liu et al., 2022c), (Liu et al., 2022d). The risk signature for predicting survival was assessed by the AUC value. We calculated the median score of risk score, which is used to select high-risk and low-risk groups in each sets. Kaplan–Meier survival analysis suggested the difference in high-risk and low-risk groups. To better evaluate clinical applications, we calculated the net benefit rates of different clinical characteristics by the DCA curve analysis.

### miRNA–mRNA Network

miRDB (Chen and Wang, 2020), TargetScan (Riffo-Campos et al., 2016), and MiTarBase (Huang et al., 2020) online tools were used to predict the downstream target genes of miRNAs significantly related to prognosis, and only genes co-existing in three databases could be selected as the ultimate target genes. Finally, Cytoscape software was used to demonstrate the mRNA–target genes network.

### Enrichment Analysis

GO enrichment analysis is a commonly used bioinformatics method, which is used to search for comprehensive information of large-scale genetic data. Meanwhile, the KEGG pathway enrichment analysis is widely used to understand biological mechanisms. The enrichment analysis was performed in downstream mRNAs by using “ggplot2” and “clusterProfiler” packages in R software (Yu et al., 2012).

### Cell Culture and qRT-PCR

As seen in a previous study (Chen et al., 2020), HCC cell lines HUH-7 and SK-HEP-1 from humans and a normal human liver cell line THLE-3 were purchased from Shanghai Institute of Cell Biology and have been identified by STR genotyping test. These cells in a culture room (5% CO2 and 37°C) were cultured using RPMI-1640 medium with 10% FBS. We used miRcute miRNA Isolation Kit (Zhisheng, Nanjing, China) to isolate total miRNA. For miRNA, miRcute Plus miRNA First-Strand cDNA Synthesis kits (Zhisheng, Nanjing, China) were used for reverse transcription. The second step was completed using miRcute Plus miRNA qPCR Detection Kits (Zhisheng, Nanjing, China). Small RNA RNU6B (U6) (RiboBio, Guangzhou, China) was used as a control for the expression of miRNA (Li et al., 2022a). Primer sequences are summarized in previous studies, including miR-139-5p (Li et al., 2022b), hsa-miR-326 (Wei et al., 2022), miR-10b-5p (Niu et al., 2021), miR-500a-3p (Long et al., 2022), and miR-592 (Paul et al., 2021).

### Statistical Analysis

All statistical analyses were performed using the R software (v.4.0.1). Detailed statistical methods about transcriptome data are covered in the bioinformatics method section. For the symbols, ∗∗∗, ∗∗, ∗, and ns, refer to *p* < 0.001, <0.01, <0.05, and not significant, respectively.

## Results

### Screening of Specific Necroptosis-Related MicroRNAs in Hepatocellular Carcinoma

**FIGURE 1:**
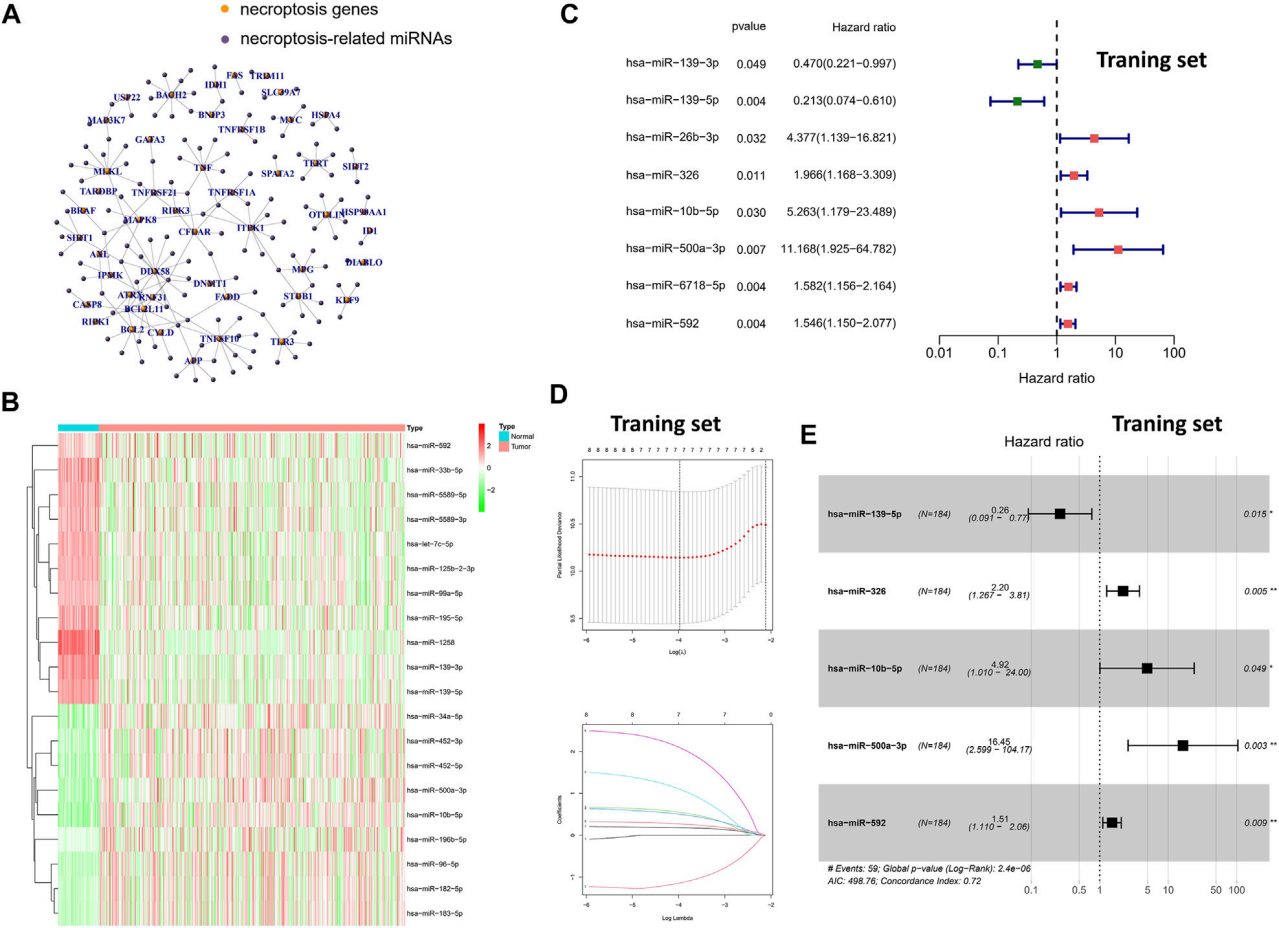
Screening of specific necroptosis-related miRNAs. **(A)** Pearson correlation analysis on 2435 miRNAs and 67 necroptosis-related genes. **(B)** The heatmap of DEms in different tissues samples. **(C)** Univariate Cox regression analysis of 35-miRNAs (only the 8 miRNAs with statistical significance were shown). **(D)** LASSO regression analysis. **(E)** Multivariate Cox regression analysis in 8 prognostic miRNAs.

Based on previous studies and Pearson correlation analysis, we obtained 144 miRNAs associated with necroptosis (Figure 1A). Subsequently, we performed differential expression analysis of the aforementioned miRNAs in the TCGA-LIHC cohort by “limma” package. Finally, a total of 35 necroptosis-related miRNAs were identified in 50 normal samples and 375 tumor samples (Figure 1B). Compared with normal tissues, the aforementioned 35-miRNAs were abnormally expressed in HCC tissues, indicating that these miRNAs were worthy of further exploration. Subsequently, univariate cox regression was performed for further screening of 35-miRNAs (Figure 1C), and LASSO regression analysis further eliminated the redundant genes in 8 prognostic miRNAs (Figure 1D). Finally, multivariate COX regression analysis was performed for the aforementioned eight prognostic miRNAs, and the minimum Akaike information criterion (AIC) value was reached when miR-139-5p, hsa-miR-326, miR-10b-5p, miR-500a-3p, and miR-592 were included in the regression equation (Figure 1E).

### A Novel Signature Based on 5-Specific Necroptosis-Related miRNAs

We developed a novel signature based on the regression coefficients of 5-specific necroptosis-related miRNAs (Table 2), and all patients were divided into high-risk and low-risk groups according to the median value of the risk score in the training set. Hence, the formula of risk score = (−1.3322 × expression level of hsa-miR-139-5p) + (0.7874 × expression level of hsa-miR-326) + (1.5938 × expression level of hsa-miR-10b-5p) + (2.8005 × expression level of hsa-miR-500a-3p) + (−0.4140 × expression level of hsa-miR-592). It is worth mentioning that we also performed Kaplan–Meier analysis and log-rank test on 5-miRNAs in the whole set (Supplementary Figure S1). The results showed that low expression of the mir-139-5p group had less possibility of survival (*p* < 0.05). Taken together, our data showed that miR-139-5p, hsa-miR-326, miR-500a-3p, and miR-592 may have potential implications for the survival of HCC patients in the whole set.

**TABLE 2:** Results of multivariate Cox regression in 5-necroptosis-related miRNAs.

miRNAs	Coef	HR	HR.95L	HR.95H	*p*
hsa-miR-139-5p	−1.3322	0.2639	0.0906	0.7687	0.0146
hsa-miR-326	0.7874	2.1976	1.2674	3.8105	0.0051
hsa-miR-10b-5p	1.5938	4.9225	1.0097	23.9995	0.0486
hsa-miR-500a-3p	2.8005	16.4536	2.5987	104.1749	0.0029
hsa-miR-592	0.4140	1.5128	1.1095	2.0627	0.0089

### Clinical Benefits of Using the Novel Signature in Patients With Hepatocellular Carcinoma

**FIGURE 2:**
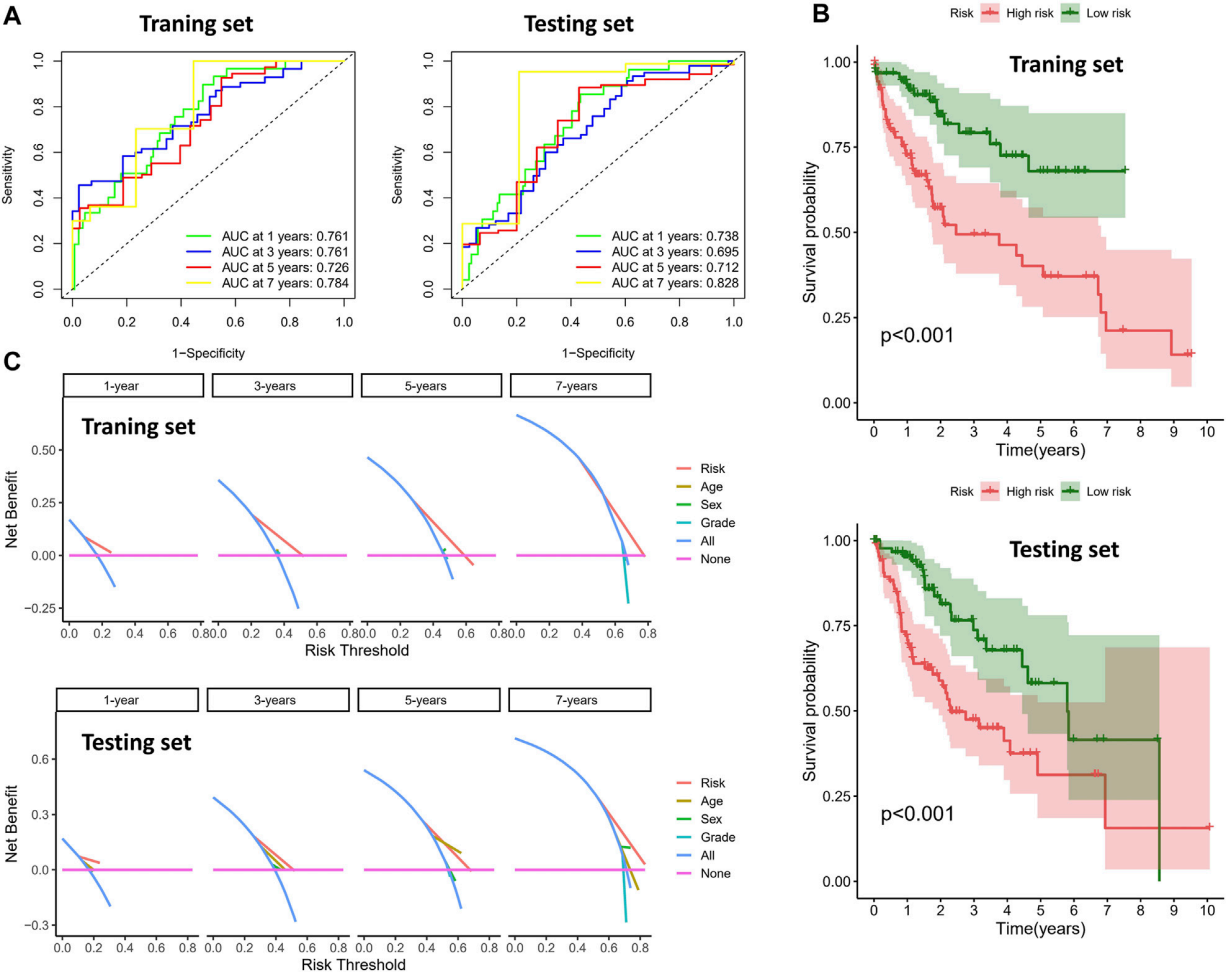
Clinical benefits using novel signature. **(A)** ROC analysis in different sets (the left figure is the training set and the right figure is the testing set). **(B)** Survival analysis of different risk groups (the top figure is the training set and the bottom figure is the testing set). **(C)** DCA analysis in different sets (the top figure is the training set and the bottom figure is the testing set).

**FIGURE 3:**
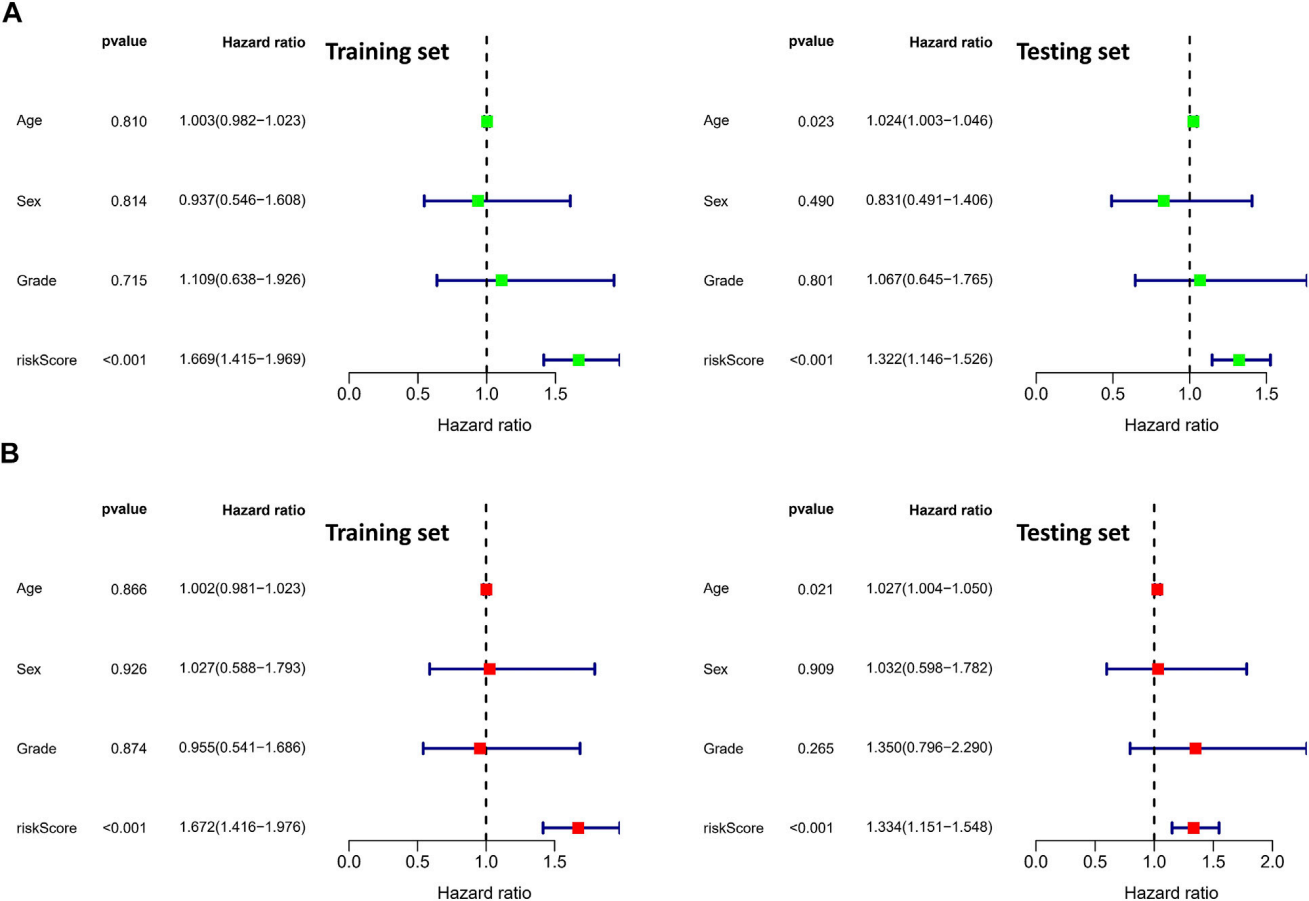
Identification of independent prognostic factors. **(A)** Univariate Cox analysis in clinical features and risk score (the left figure is the training set and the right figure is the testing set). **(B)** Multivariate Cox analysis in clinical features and risk score (the left figure is the training set and the right figure is the testing set).

**FIGURE 4:**
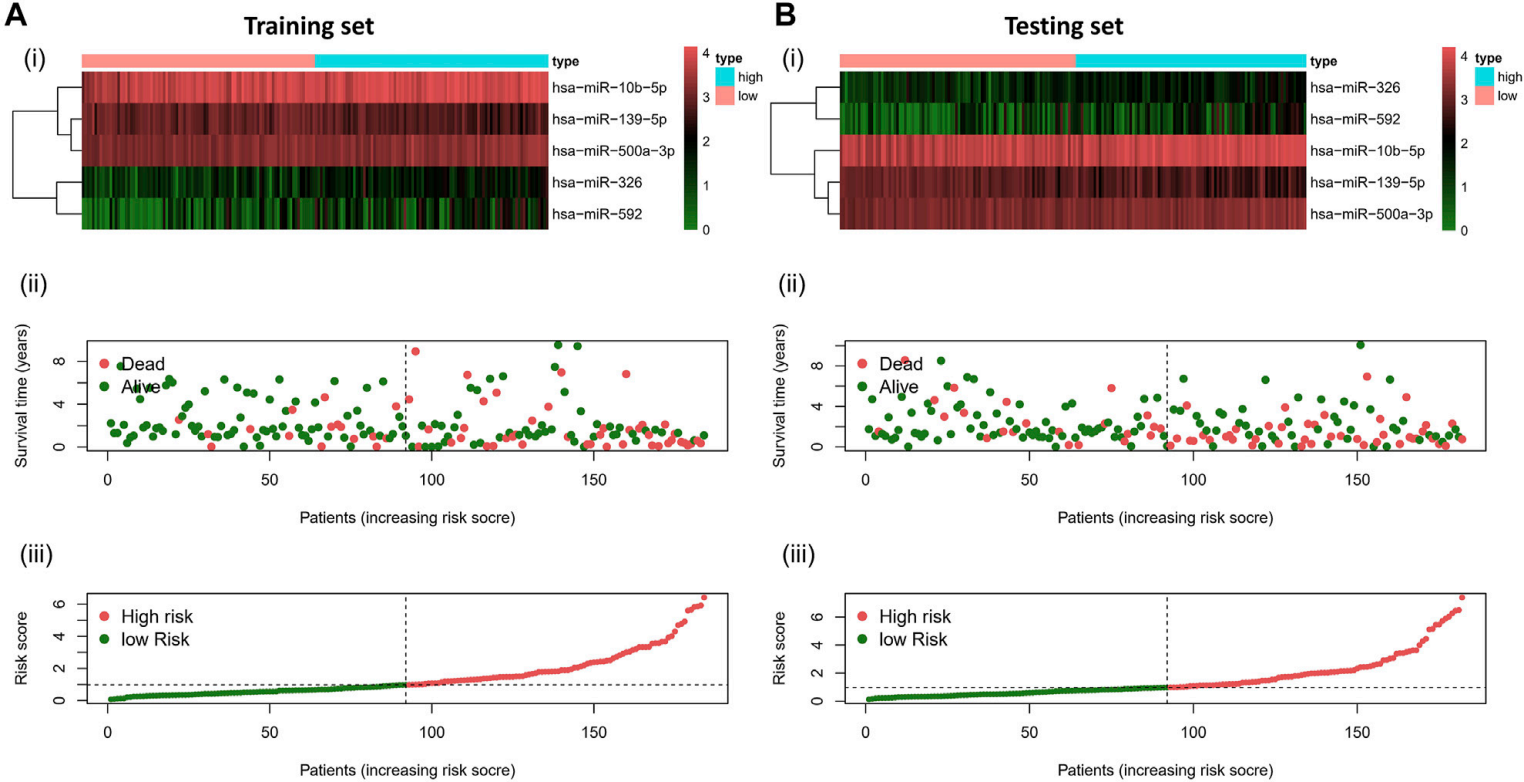
Risk distribution of all patients. **(A)**Training sets **[(A), i]** Heatmap of different risk groups in 5-specific necroptosis-related miRNAs. **[(A), ii]** Risk score scatter plot. **[(A), iii]** Risk score curve plot. **(B)** Testing sets **[(B), i]** Heatmap of different risk group in 5-specific necroptosis-related miRNAs. **[(B), ii]** Risk score scatter plot. **[(A), iii]** Risk score curve plot. Patients are categorized into low-risk (green) and high-risk (red) groups.

To validate the prognostic value of the risk score, we conducted survival analyses by plotting Kaplan–Meier curves and ROC analysis. As a result, the area under curves (AUCs) of 1 year, 3 years, 5 years, and 7 years were high in the training and testing sets (AUC = 0.761, 0.761, 0.726, and 0.784 in the training set; AUC = 0.738, 0.695, 0.712, and 0.828 in the testing set), as shown in Figure 2A. In addition, the survival rate can be separated between the high-risk and low-risk groups, and the high-risk group showed low survival probabilities (*p* < 0.001), as shown in Figure 2B. Interestingly, DCA analysis also revealed that the risk score had a net benefit value than other clinical characteristics (Figure 2C). To validate whether the risk score, patient age, tumor grade, and gender can function as independent predictors, we conducted univariate (Figure 3A) and multivariate Cox regression (Figure 3B) analyses for these clinical characteristics in the training set and testing set. The results showed that the risk score was an independent prognosis factor among the four clinical characteristics in multivariate analysis (HR = 1.672 in training set, HR = 1.334 in testing set). The heatmap showed the expression of 5-specific necroptosis-related miRNAs in different risk groups [Figures 4Ai, Bi]. In addition, as the risk score increased, the patient death risk increased and the survival time decreased [Figure 4Aii,iii, Bii,iii].

### Construction of Regulatory Network

**FIGURE 5:**
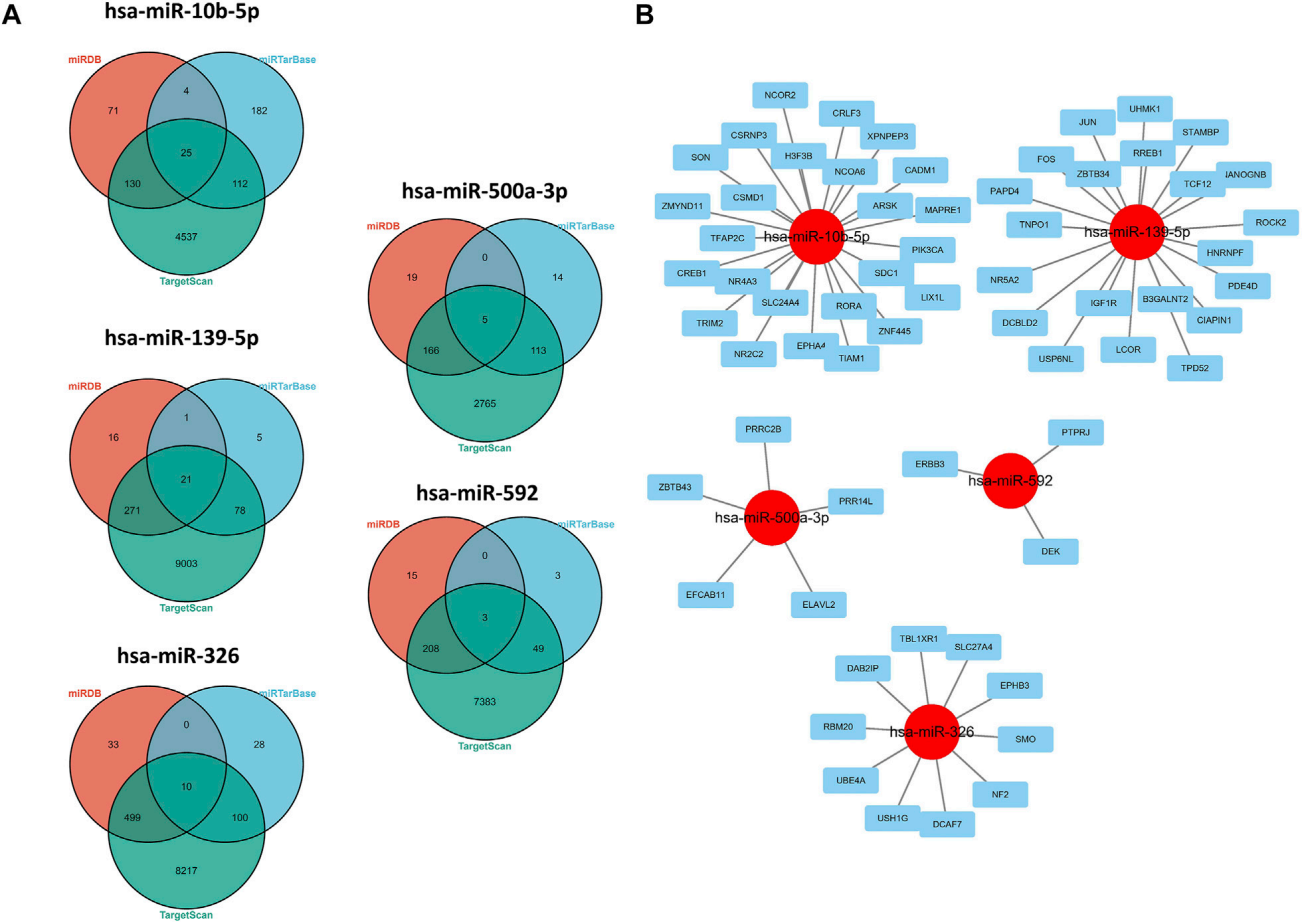
Regulatory network of 5-miRNAs. **(A)** Venn plots in three databases. **(B)** Regulatory network conducted by Cytoscape software.

As shown in the results of the aforementioned section, miR-139-5p, hsa-miR-326, miR-10b-5p, miR-500a-3p, and miR-592 may have potential implications for survival of HCC patients, so we explored the potential regulatory axis using miRDB, TargetScan, and MiTarBase online tools. Finally, 21, 10, 25, 5, and 3 potential downstream targeted genes were identified in miR-139-5p, hsa-miR-326, miR-10b-5p, miR-500a-3p, and miR-592, respectively (Figure 5A). As shown in Figure 5B, we visualized the aforementioned network. More specifically, ARSK, CADM1, EPHA4, H3F3B, MAPRE1, NCOA6, NCOR2, NR2C2, NR4A3, PIK3CA, RORA, CREB1, CRLF3, CSMD1, CSRNP3, SLC24A4, SON, TFAP2C, TIAM1, TRIM2, XPNPEP3, ZMYND11, ZNF445, LIX1L, and SDC1 were the downstream targets of miR-10b-5p. ZBTB34, UHMK1, USP6NL, CIAPIN1, TPD52, IGF-1R, STAMBP, TCF12, DCBLD2, TNPO1, B3GALNT2, NANOGNB, NR5A2, PAPD4, PDE4D, ROCK2, RREB1, JUN, FOS, HNRNPF, and LCOR were the downstream targets of miR-139-5p. UBE4A, USH1G, DAB2IP, DCAF7, EPHB3, NF2, RBM20, SLC27A4, SMO, and TBL1XR1 were the downstream targets of miR-326. ZBTB43, PRR14L, PRRC2B, EFCAB11, and ELAVL2 were the downstream targets of miR-500a-3p. ERBB3, DEK, and PTPRJ were the downstream targets of miR-592.

### Gene Enrichment Analysis

**FIGURE 6:**
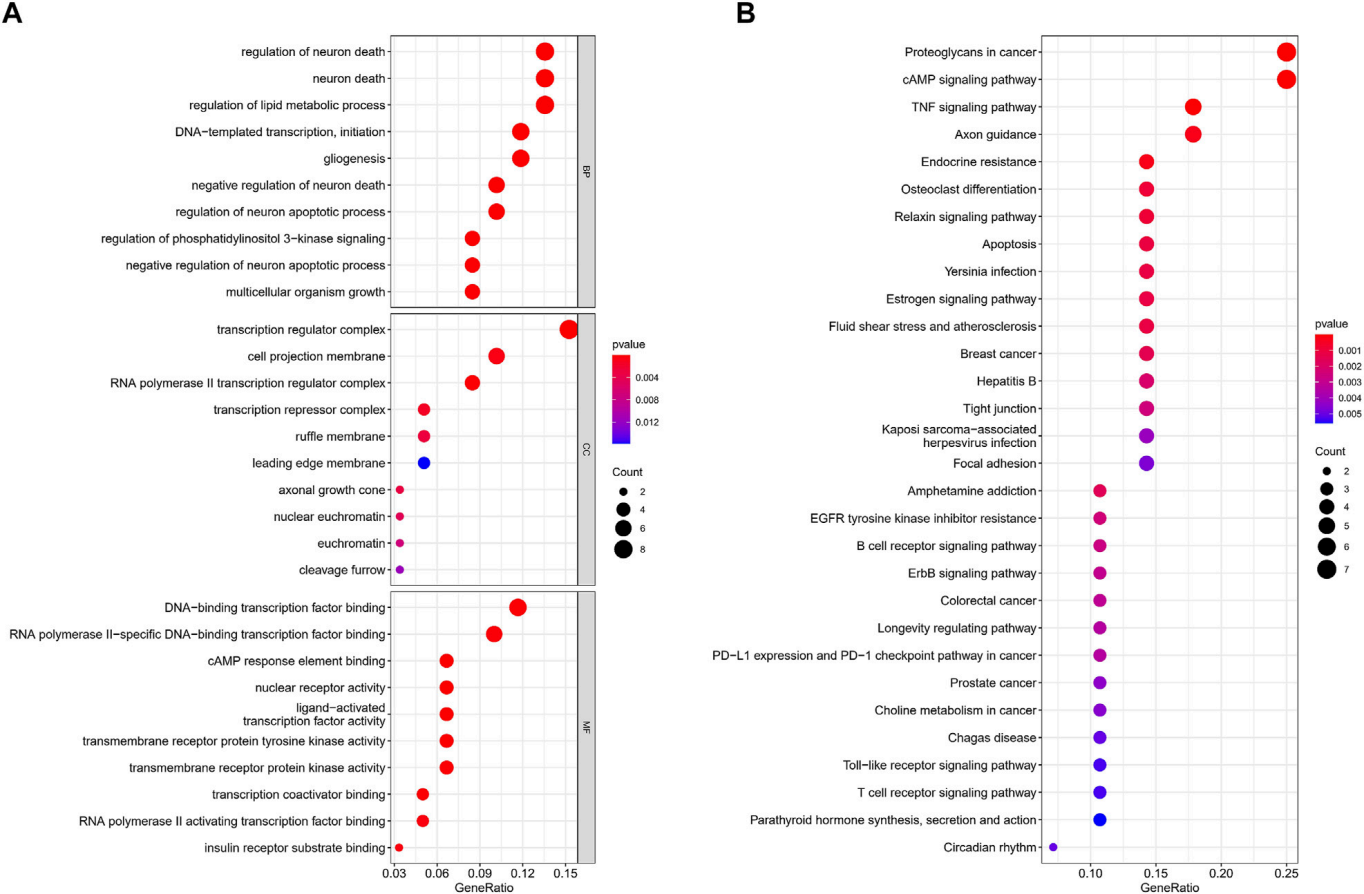
GO and KEGG enrichment analysis. **(A)** GO analysis. **(B)** KEGG analysis.

In order to further explore the biological functions involved in necroptosis-related miRNAs, we conducted an in-depth enrichment analysis. The results of GO enrichment analysis of the aforementioned targeted genes showed that they were mainly enriched in the regulation of neuron death, DNA-templated transcription, and gliogenesis. (Figure 6A). Meanwhile, KEGG enrichment further elucidated possible pathways such as proteoglycans in cancer, cAMP signaling pathway, and TNF signaling pathway. (Figure 6B).

### qRT-PCR

**FIGURE 7:**
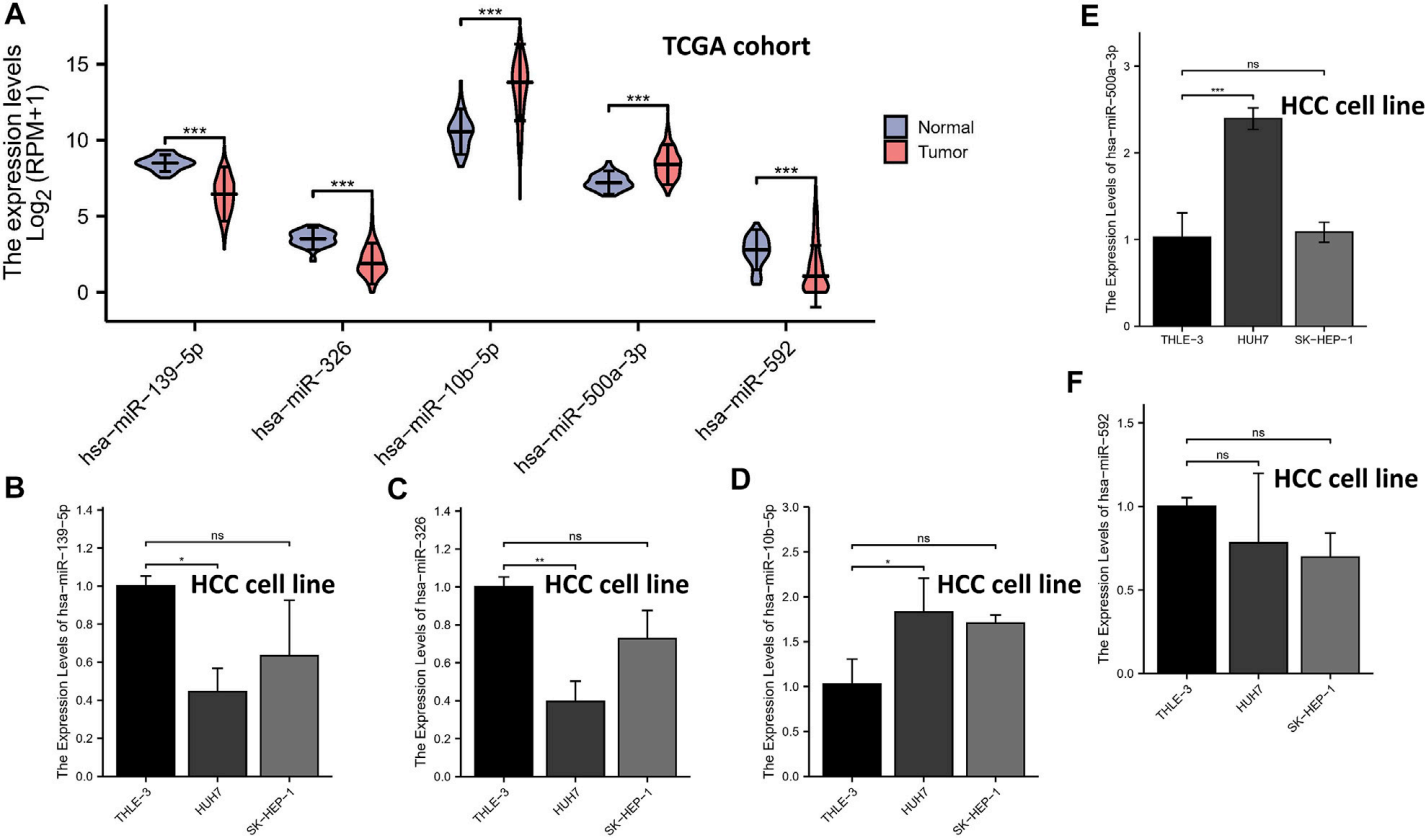
qRT-PCR. The expression levels of the 5-miRNAs in the TCGA database **(A)**. The expression levels of miR-139-5p **(B)**, hsa-miR-326 **(C)**, miR-10b-5p **(D)**, miR-500a-3p **(E)**, and miR-592 **(F)**.

The expression levels of the 5-miRNAs were determined using the TCGA database and qRT-PCR experiment. As shown in Figure 7A, the violin plot showed that tumor tissues had higher expression levels of miR-10b-5p and miR-500a-3p than normal tissues, while normal samples had higher expression levels of miR-139-5p, hsa-miR-326, and miR-592 compared to HCC samples. Subsequently, HCC cell lines (HUH-7 and SK-HEP-1) and a normal human liver cell line (THLE-3) were used to validate the results of tissue samples. In the validation of miR-139-5p (Figure 7B), hsa-miR-326 (Figure 7C), miR-10b-5p (Figure 7D), and miR-500a-3p (Figure 7E), the expression of HUH-7 was higher than that of THLE-3. However, there was no statistical difference in the expression of the aforementioned miRNAs in SK-HEP-1 vs. THLE-3. In the validation of miR-592, no significant differences in the expression of all three cell lines were observed (Figure 7F). Overall, most miRNA expressions were at the same level in cell lines and tissues, except for miR-592.

## Discussion

Although there are many studies on miRNA expression to predict the prognosis of patients, no study has systematically used necroptosis-related miRNA to predict the prognosis of HCC patients. To our knowledge, our study is the first to explore the clinical application of necroptosis-related miRNAs in an HCC cohort. In this study, a total of 5-miRNA signatures were generated by Cox regression. Subsequently, a series of statistical analyses demonstrated the excellent clinical application for the risk score calculated based on the risk signature. Overall, most miRNA expressions were at the same level in cell lines and tissues, except for miR-592. Moreover, we found that specific miRNAs were mainly enriched in the cAMP signaling pathway, TNF signaling pathway, and Wnt/β-catenin pathway. Fortunately, the role of the aforementioned pathways in the pathogenesis of HCC has been verified in *in vitro* experiments. Liu et al. (2021b) revealed that cellular retinol binding protein-1 inhibits cancer stemness *via* upregulating WIF1 to suppress the Wnt/β-catenin pathway in hepatocellular carcinoma. Meanwhile, Yassin et al. (2022) explored silybum marianum total extract, silymarin and silibinin abate hepatocarcinogenesis, and hepatocellular carcinoma growth *via* modulation of the TNF signaling pathway. In addition, activation of the cAMP signaling pathway in HCC also appeared to be associated with epigenetic modifications, as in Cai et al.’s (2021) study that RBM15-mediated m6A modification might facilitate the progression of HCC *via* the IGF2BP1-YES1-MAPK axis. This seems to suggest that there is cross-talk in the pathways that promote the development of HCC. Thus, this study lays the foundation for our future *in vivo* and *in vitro* experiments.

Most of the miRNAs participating in signatures have been revealed to be associated with cancer progression. In miR-139-5p, it suppressed the proliferation and migration of hepatocellular carcinoma cells by downregulating the expression of ENAH (Zhang et al., 2022). Meanwhile, miR-326 can suppresses the Hippo pathway when combined with PAX8 in trophoblast (Zang et al., 2022). Moreover, Wang and Chen (2022) also revealed that overexpression of miR-362 inhibited the expression of JMJD2A in nasopharyngeal carcinoma, and aberrant miR-362 may be associated with EBV-infection. In miR-10b-5p, it can inhibit tumorigenesis in the gastric cancer xenograft mice model by downregulating Tiam1 (Liu et al., 2021c); therefore, Yan et al. (2021) put forward a novel idea for exosomal miR-10b-5p in gastric cancer. A novel conclusion was that upregulated exosomal miR-10b-5p is involved in fibroblasts in tumor microenvironment. In miR-500a-3p, it has been shown that silence miR-500a-3p may serve as a new therapeutic strategy in the treatment of HCC (Jiang et al., 2017). Currently, there are three studies on the mechanism of miR-592 in HCC, in which the downstream consists of IGF-1R (Wang et al., 2017b), WSB1 (Jia et al., 2016), and DEK (Li et al., 2015). In addition to the miRNAs involved in the model, other miRNAs have also been studied in depth in HCC. In some studies about miR-223-3p, it can be used as a novel noninvasive biomarker for HCV-positive cirrhosis and HCC (Oksuz et al., 2015). Circulating mirR-223-3p can represent novel diagnostic and prognostic markers for HBV-associated HCC patients (Pratedrat et al., 2020). It is interesting to note that miR-331-3p (AUC: 0.832) has better diagnostic performance than AFP (Jin et al., 2019). On the other hand, these evidence suggest that sequencing of blood samples from HCC patients may play a noninvasive role in predicting survival outcomes. The novel mechanism of miR-500a-3p promoting HCC stem cell maintenance suggests that miR-500a-3p may be a novel therapeutic strategy (Jiang et al., 2017). Unfortunately, the critical role of the aforementioned pathways in HCC necroptosis is not well explained at present.

However, this study only used the data from the public database TCGA to construct the model, and there was no condition to collect our data to validate 5-miRNA signature, which was a limitation to our study. In addition, we have no conditions to verify the mechanism of these necroptosis-related miRNAs. In the future, we can use risk signatures based on altered 5-miRNAs, which may improve prognostic prediction and may lead to development of targeted therapy.

## Conclusion

Comprehensively, our study suggests that necroptosis-related miRNAs is closely associated with the prognosis of HCC, and we established a robust tool for the prognostic management of HCC patients.

## Data Availability

Publicly available datasets were analyzed in this study. These data can be found at: https://portal.gdc.cancer.gov/.

## Supplementary Material

Supplementary Figure S1 Kaplan–Meier analysis about 5-specific necroptosis-related miRNAs in the whole set. miR-10b-5p **(A)**, miR-139-5p **(B)**, hsa-miR-326 **(C)**, miR-500a-3p **(D)**, and miR-592 **(E)**.

## References

[B1] Bartel D. P. (2004). MicroRNAs. Cell 116 (2), 281–297. 10.1016/s0092-8674(04)00045-5 14744438

[B2] Cai X. Chen Y. Man D. Yang B. Feng X. Zhang D. (2021). RBM15 Promotes Hepatocellular Carcinoma Progression by Regulating N6-Methyladenosine Modification of YES1 mRNA in an IGF2BP1-dependent Manner. Cell Death Discov. 7, 315. 10.1038/s41420-021-00703-w 34707107 PMC8551180

[B3] Chen B. Liao Z. Qi Y. Zhang H. Su C. Liang H. (2020). miR-631 Inhibits Intrahepatic Metastasis of Hepatocellular Carcinoma by Targeting PTPRE. Front. Oncol. 10, 565266. 10.3389/fonc.2020.565266 33344226 PMC7746836

[B4] Chen Y. Wang X. (2020). miRDB: an Online Database for Prediction of Functional microRNA Targets. Nucleic Acids Res. 48 (D1), D127–D131. 10.1093/nar/gkz757 31504780 PMC6943051

[B5] Christofferson D. E. Yuan J. (2010). Necroptosis as an Alternative Form of Programmed Cell Death. Curr. Opin. Cel Biol. 22 (2), 263–268. 10.1016/j.ceb.2009.12.003 PMC2854308 20045303

[B6] Di Sandro S. Centonze L. Centonze L. Pinotti E. Lauterio A. De Carlis R. (2019). Surgical and Oncological Outcomes of Hepatic Resection for BCLC-B Hepatocellular Carcinoma: a Retrospective Multicenter Analysis Among 474 Consecutive Cases. Updates Surg. 71 (2), 285–293. 10.1007/s13304-019-00649-w 30941704

[B7] Ding B. Fan W. Lou W. (2020). hsa_circ_0001955 Enhances *In Vitro* Proliferation, Migration, and Invasion of HCC Cells through miR-145-5p/NRAS Axis. Mol. Ther. - Nucleic Acids 22, 445–455. 10.1016/j.omtn.2020.09.007 33230448 PMC7554323

[B8] Dionísio P. A. Amaral J. D. Rodrigues C. M. P. (2020). Molecular Mechanisms of Necroptosis and Relevance for Neurodegenerative Diseases. Int. Rev. Cel Mol Biol 353, 31–82. 10.1016/bs.ircmb.2019.12.006 32381178

[B9] El-Serag H. B. (2011). Hepatocellular Carcinoma. N. Engl. J. Med. 365 (12), 1118–1127. 10.1056/NEJMra1001683 21992124

[B10] Gong Y. Fan Z. Luo G. Yang C. Huang Q. Fan K. (2019). The Role of Necroptosis in Cancer Biology and Therapy. Mol. Cancer 18. 100. 10.1186/s12943-019-1029-8 31122251 PMC6532150

[B11] Hitomi J. Christofferson D. E. Ng A. Yao J. Degterev A. Xavier R. J. (2008). Identification of a Molecular Signaling Network that Regulates a Cellular Necrotic Cell Death Pathway. Cell 135 (7), 1311–1323. 10.1016/j.cell.2008.10.044 19109899 PMC2621059

[B12] Hu T. Zhao X. Zhao Y. Cheng J. Xiong J. Lu C. (2022). Identification and Verification of Necroptosis-Related Gene Signature and Associated Regulatory Axis in Breast Cancer. Front. Genet. 13, 842218. 10.3389/fgene.2022.842218 35251139 PMC8888972

[B13] Huang H.-Y. Lin Y.-C. -D. Li J. Huang K.-Y. Shrestha S. Hong H.-C. (2020). miRTarBase 2020: Updates to the Experimentally Validated microRNA-Target Interaction Database. Nucleic Acids Res. 48 (D1), D148–D154. 10.1093/nar/gkz896 31647101 PMC7145596

[B14] Huang Y. Zou Y. Xiong Q. Zhang C. Sayagués J. M. Shelat V. G. (2021). Development of a Novel Necroptosis-Associated miRNA Risk Signature to Evaluate the Prognosis of colon Cancer Patients. Ann. Transl Med. 9 (24), 1800. 10.21037/atm-21-6576 35071494 PMC8756225

[B15] Jia Y.-Y. Zhao J.-Y. Li B.-L. Gao K. Song Y. Liu M.-Y. (2016). miR-592/WSB1/HIF-1α axis Inhibits Glycolytic Metabolism to Decrease Hepatocellular Carcinoma Growth. Oncotarget 7 (23), 35257–35269. 10.18632/oncotarget.9135 27153552 PMC5085226

[B16] Jiang C. Long J. Liu B. Xu M. Wang W. Xie X. (2017). miR-500a-3p Promotes Cancer Stem Cells Properties via STAT3 Pathway in Human Hepatocellular Carcinoma. J. Exp. Clin. Cancer Res. 36, 99. 10.1186/s13046-017-0568-3 28750679 PMC5532790

[B17] Jin W. Zhong N. Wang L. Yu J. Yin F. Zhang K. (2019). MiR-331-3p Inhibition of the Hepatocellular Carcinoma (HCC) Bel-7402 Cell Line by Down-Regulation of E2F1. J. Nanosci Nanotechnol 19 (9), 5476–5482. 10.1166/jnn.2019.16535 30961699

[B18] Lalaoui N. Lindqvist L. M. Sandow J. J. Ekert P. G. (2015). The Molecular Relationships between Apoptosis, Autophagy and Necroptosis. Semin. Cel Develop. Biol. 39, 63–69. 10.1016/j.semcdb.2015.02.003 25736836

[B19] Li J. McQuade T. Siemer A. B. Napetschnig J. Moriwaki K. Hsiao Y.-S. (2012). The RIP1/RIP3 Necrosome Forms a Functional Amyloid Signaling Complex Required for Programmed Necrosis. Cell 150 (2), 339–350. 10.1016/j.cell.2012.06.019 22817896 PMC3664196

[B20] Li W. Kong X. Huang T. Shen L. Wu P. Chen Q.-F. (2020). Bioinformatic Analysis and *In Vitro* Validation of a Five-microRNA Signature as a Prognostic Biomarker of Hepatocellular Carcinoma. Ann. Transl Med. 8 (21), 1422. 10.21037/atm-20-2509 33313167 PMC7723630

[B21] Li X. Zhang W. Zhou L. Yue D. Su X. (2015). MicroRNA-592 Targets DEK Oncogene and Suppresses Cell Growth in the Hepatocellular Carcinoma Cell Line HepG2. Int. J. Clin. Exp. Pathol. 8, 12455 26722432 PMC4680377

[B22] Li X. Ren Y. Liu D. Yu X. Chen K. (2022a). Role of miR-100-5p and CDC25A in Breast Carcinoma Cells. PeerJ 9, e12263. 10.7717/peerj.12263 35036112 PMC8734459

[B23] Li Y. Sun Y. Li Z. Li S. Wu C. (2022b). MiR-139-5p Inhibits the Development of Gastric Cancer through Targeting TPD52. J. Healthc. Eng. 2022, 4033373. 10.1155/2022/4033373 35222884 PMC8866006

[B24] Linkermann A. Green D. R. (2014). Necroptosis. N. Engl. J. Med. 370 (5), 455–465. 10.1056/NEJMra1310050 24476434 PMC4035222

[B25] Liu F. An X. Zhao X. Zhang N. Chen B. Li Z. (2021c). MiR-10b-5p Inhibits Tumorigenesis in Gastric Cancer Xenograft Mice Model through Down-Regulating Tiam1. Exp. Cel Res. 407 (2), 112810. 10.1016/j.yexcr.2021.112810 34487733

[B26] Liu X. Shan W. Li T. Gao X. Kong F. You H. (2021b). Cellular Retinol Binding Protein-1 Inhibits Cancer Stemness via Upregulating WIF1 to Suppress Wnt/β-Catenin Pathway in Hepatocellular Carcinoma. BMC Cancer 21 (1), 1224. 10.1186/s12885-021-08967-2 34775955 PMC8590789

[B27] Liu Y. Chen Q. Zhu Y. Wang T. Ye L. Han L. (2021a). Non-coding RNAs in Necroptosis, Pyroptosis and Ferroptosis in Cancer Metastasis. Cel Death Discov. 7, 210. 10.1038/s41420-021-00596-9 PMC8358062 34381023

[B28] Liu Z. Guo C. Dang Q. Wang L. Liu L. Weng S. (2022b). Integrative Analysis from Multi-center Studies Identities a Consensus Machine Learning-Derived lncRNA Signature for Stage II/III Colorectal Cancer. EBioMedicine 75, 103750. 10.1016/j.ebiom.2021.103750 34922323 PMC8686027

[B29] Liu Z. Guo Y. Yang X. Chen C. Fan D. Wu X. (2022d). Immune Landscape Refines the Classification of Colorectal Cancer with Heterogeneous Prognosis, Tumor Microenvironment and Distinct Sensitivity to Frontline Therapies. Front. Cel Dev. Biol. 9, 784199. 10.3389/fcell.2021.784199 PMC8784608 35083217

[B30] Liu Z. Liu L. Weng S. Guo C. Dang Q. Xu H. (2022a). Machine Learning-Based Integration Develops an Immune-Derived lncRNA Signature for Improving Outcomes in Colorectal Cancer. Nat. Commun. 13, 816. 10.1038/s41467-022-28421-6 35145098 PMC8831564

[B31] Liu Z. Xu H. Weng S. Ren Y. Han X. (2021c). Stemness Refines the Classification of Colorectal Cancer with Stratified Prognosis, Multi-Omics Landscape, Potential Mechanisms, and Treatment Options. Front. Immunol. 13, 828330. 10.3389/fimmu.2022.828330 PMC8828967 35154148

[B32] Llovet J. M. Kelley R. K. Villanueva A. Singal A. G. Pikarsky E. Roayaie S. (2021). Hepatocellular Carcinoma. Nat. Rev. Dis. Primers 7. 10.1038/s41572-020-00240-3 PMC13537433 33479224

[B33] Long J. Liu B. Yao Z. Weng H. Li H. Jiang C. (2022). miR-500a-3p Is a Potential Prognostic Biomarker in Hepatocellular Carcinoma. Ijgm 15:1891–1899. 10.2147/IJGM.S340629 PMC8881010 35221718

[B34] Long J. S. Ryan K. M. (2012). New Frontiers in Promoting Tumour Cell Death: Targeting Apoptosis, Necroptosis and Autophagy. Oncogene 31 (49), 5045–5060. 10.1038/onc.2012.7 22310284

[B35] Niu X. Sun H. Qiu F. Liu J. Yang T. Han W. (2021). miR-10b-5p Suppresses the Proliferation and Invasion of Primary Hepatic Carcinoma Cells by Downregulating EphA2. Biomed. Res. Int. 2021, 1382061. 10.1155/2021/1382061 35005012 PMC8731268

[B36] Oksuz Z. Serin M. S. Kaplan E. Dogen A. Tezcan S. Aslan G. (2015). Serum microRNAs; miR-30c-5p, miR-223-3p, miR-302c-3p and miR-17-5p Could Be Used as Novel Non-invasive Biomarkers for HCV-Positive Cirrhosis and Hepatocellular Carcinoma. Mol. Biol. Rep. 42 (3), 713–720. 10.1007/s11033-014-3819-9 25391771

[B37] Paul R. Bapat P. Deogharkar A. Kazi S. Singh S. K. V. Gupta T. (2021). MiR-592 Activates the mTOR Kinase, ERK1/ERK2 Kinase Signaling and Imparts Neuronal Differentiation Signature Characteristic of Group 4 Medulloblastoma. Hum. Mol. Genet. 30 (24), 2524–2525. 10.1093/hmg/ddab264 34554250

[B38] Pratedrat P. Chuaypen N. Nimsamer P. Payungporn S. Pinjaroen N. Sirichindakul B. (2020). Diagnostic and Prognostic Roles of Circulating miRNA-223-3p in Hepatitis B Virus-Related Hepatocellular Carcinoma. PLoS One 15 (4), e0232211. 10.1371/journal.pone.0232211 32330203 PMC7182200

[B39] Riffo-Campos Á. Riquelme I. Brebi-Mieville P. (2016). Tools for Sequence-Based miRNA Target Prediction: What to Choose? Ijms 17 (12), 1987. 10.3390/ijms17121987 PMC5187787 27941681

[B40] Ritchie M. E. Phipson B. Wu D. Hu Y. Law C. W. Shi W. (2015). Limma powers Differential Expression Analyses for RNA-Sequencing and Microarray Studies. Nucleic Acids Res. 43 (7), e47. 10.1093/nar/gkv007 25605792 PMC4402510

[B41] Ruan Z.-h. Xu Z.-x. Zhou X.-y. Zhang X. Shang L. (2019). Implications of Necroptosis for Cardiovascular Diseases. Curr. Med. Sci. 39 (4), 513–522. 10.1007/s11596-019-2067-6 31346984

[B42] Seehawer M. Heinzmann F. D’Artista L. Harbig J. Roux P.-F. Hoenicke L. (2018). Necroptosis Microenvironment Directs Lineage Commitment in Liver Cancer Nature 562, 69–75. 10.1038/s41586-018-0519-y 30209397 PMC8111790

[B43] Strilic B. Yang L. Albarrán-Juárez J. Wachsmuth L. Han K. Müller U. C. (2016). Tumour-cell-induced Endothelial Cell Necroptosis via Death Receptor 6 Promotes Metastasis. Nature 536 (7615), 215–218. 10.1038/nature19076 27487218

[B44] Visalli M. Bartolotta M. Polito F. Oteri R. Barbera A. Arrigo R. (2018). miRNA Expression Profiling Regulates Necroptotic Cell Death in Hepatocellular Carcinoma. Int. J. Oncol. 53 (2), 771–780. 10.3892/ijo.2018.4410 29845207

[B45] Wang T. Xu L. Jia R. Wei J. (2017a). MiR-218 Suppresses the Metastasis and EMT of HCC Cells via Targeting SERBP1. Acta Biochim. Biophys. Sin (Shanghai). 49 (5), 383–391. 10.1093/abbs/gmx017 28369267

[B46] Wang W. Zhang H. Tang M. Liu L. Zhou Z. Zhang S. (2017b). MicroRNA-592 Targets IGF-1R to Suppress Cellular Proliferation, Migration and Invasion in Hepatocellular Carcinoma. Oncol. Lett. 13 (5), 3522–3528. 10.3892/ol.2017.5902 28529580 PMC5431753

[B47] Wang X. Chen P. (2022). Aberrant miR-362-3p Is Associated with EBV-Infection and Prognosis in Nasopharyngeal Carcinoma and Involved in Tumor Progression by Targeting JMJD2A. Ott 15, 121. 10.2147/OTT.S325100 PMC8806052 35115787

[B48] Wang Z. Jensen M. A. Zenklusen J. C. (2016). A Practical Guide to the Cancer Genome Atlas (TCGA). Methods Mol. Biol. 1418, 111–141. 10.1007/978-1-4939-3578-9_6 27008012

[B49] Wei B. Wang Z. Lian Q. Chi B. Ma S. (2022). hsa_circ_0139402 Promotes Bladder Cancer Progression by Regulating Hsa-miR-326/pax8 Signaling. Dis. Markers, 2022, 9899548. 10.1155/2022/9899548 35154515 PMC8824756

[B50] Xiang Y. k. Peng F. h. Guo Y. q. Ge H. Cai S. y. Fan L. x. (2021). Connexin32 Activates Necroptosis through Src‐mediated Inhibition of Caspase 8 in Hepatocellular Carcinoma. Cancer Sci. 112 (9), 3507–3519. 10.1111/cas.14994 34050696 PMC8409421

[B51] Yan T. Wang X. Wei G. Li H. Hao L. Liu Y. (2021). Exosomal miR-10b-5p Mediates Cell Communication of Gastric Cancer Cells and Fibroblasts and Facilitates Cell Proliferation. J. Cancer 12, 2140–2150. 10.7150/jca.47817 33754012 PMC7974515

[B52] Yassin N. Y. S. AbouZid S. F. El-Kalaawy A. M. Ali T. M. Almehmadi M. M. Ahmed O. M. (2022). Silybum marianum Total Extract, Silymarin and Silibinin Abate Hepatocarcinogenesis and Hepatocellular Carcinoma Growth via Modulation of the HGF/c-Met, Wnt/β-Catenin, and PI3K/Akt/mTOR Signaling Pathways. Biomed. Pharmacother. 145, 112409. 10.1016/j.biopha.2021.112409 34781148

[B53] Yu G. Wang L.-G. Han Y. He Q.-Y. (2012). clusterProfiler: an R Package for Comparing Biological Themes Among Gene Clusters. OMICS: A J. Integr. Biol. 16 (5), 284–287. 10.1089/omi.2011.0118 PMC3339379 22455463

[B54] Zang J. Yan M. Zhang Y. Peng W. Zuo J. Zhou H. (2022). MiR-326 Inhibits Trophoblast Growth, Migration, and Invasion by Targeting PAX8 via Hippo Pathway. Reprod. Biol. Endocrinol. 20, 38. 10.1186/s12958-022-00909-2 35209928 PMC8867866

[B55] Zhang Y. Li M. Qiu Y. Wu Y. Chen S. Ni B. (2022). MiR-139-5p/ENAH Affects Progression of Hepatocellular Carcinoma Cells. Biochem. Genet. [Epub ahead of print]. 10.1007/s10528-022-10204-9 35254597

[B56] Zhang Y. Qian M. Tang F. Huang Q. Wang W. Li Y. (2020). Identification and Analysis of P53-Regulated Enhancers in Hepatic Carcinoma. Front. Bioeng. Biotechnol. 8, 668, 10.3389/fbioe.2020.00668 32695760 PMC7338759

