# Supplementary figures and images for: Construction of a Necroptosis-Related miRNA Signature for Predicting the Prognosis of Patients With Hepatocellular Carcinoma
Source: Front Genet. 2022 Apr 12;13:825261. doi: 10.3389/fgene.2022.825261 (PMC9039163; doi:10.3389/fgene.2022.825261)

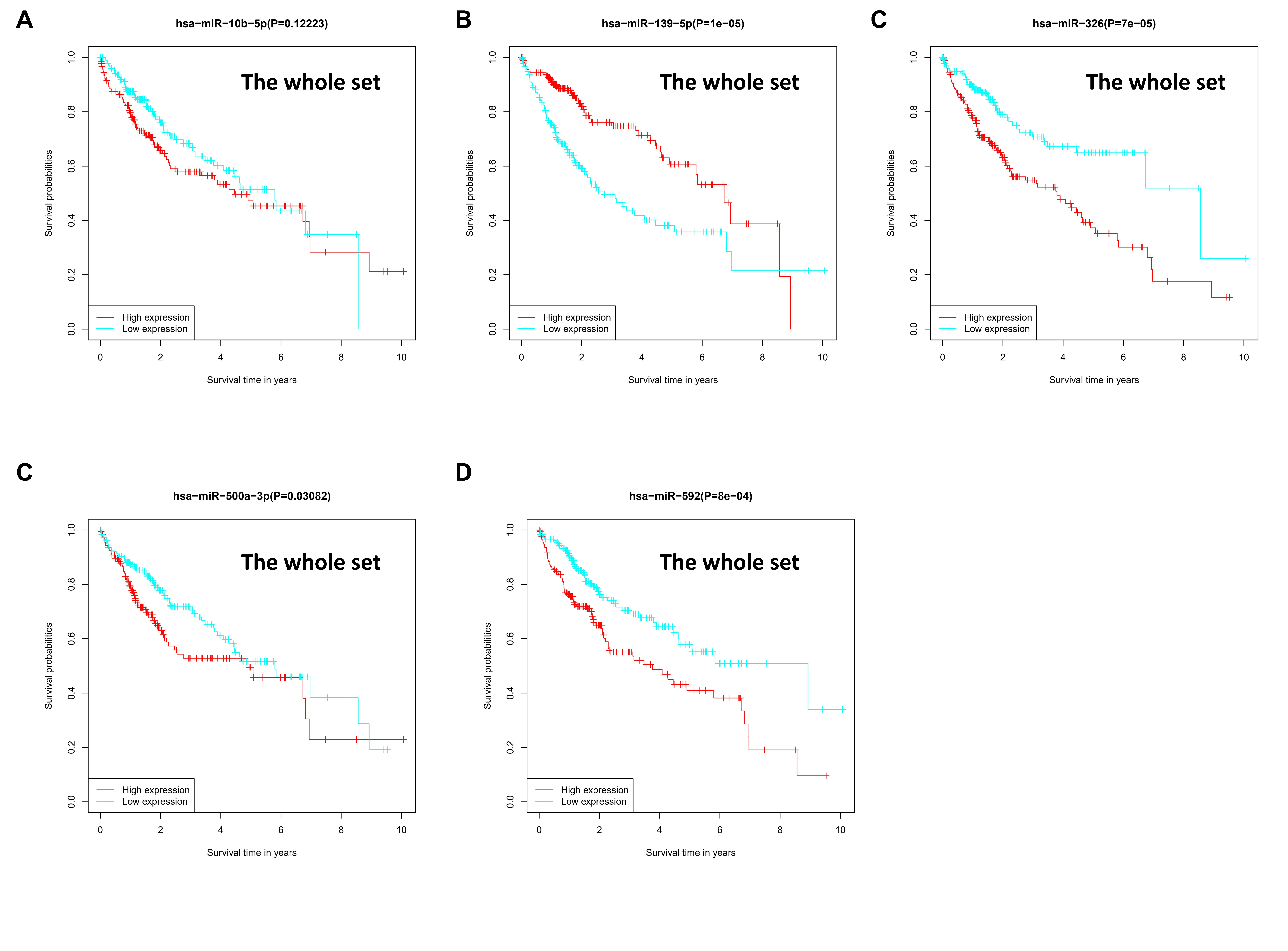

Supplement: Supplementary file 2 [file Image1.TIFF]

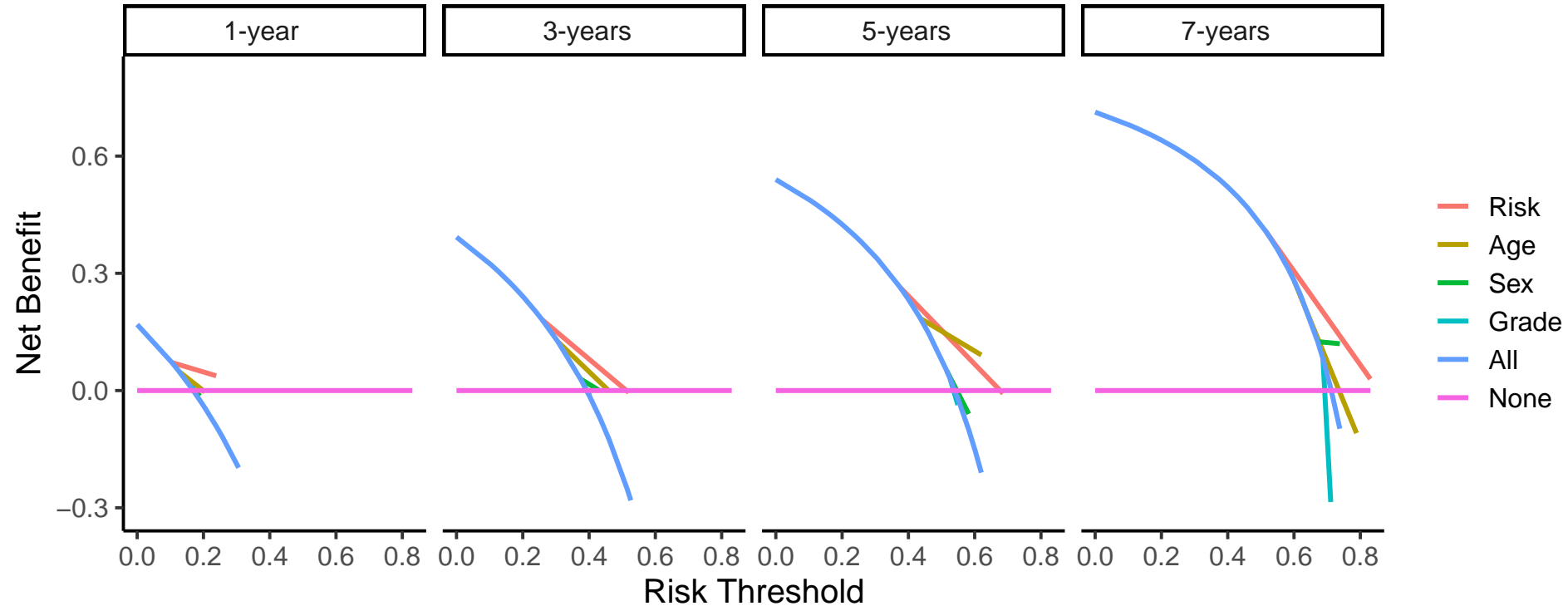

Supplement: Supplementary file 3 [file DataSheet1.ZIP › raw data/7-/DCA-test.pdf]

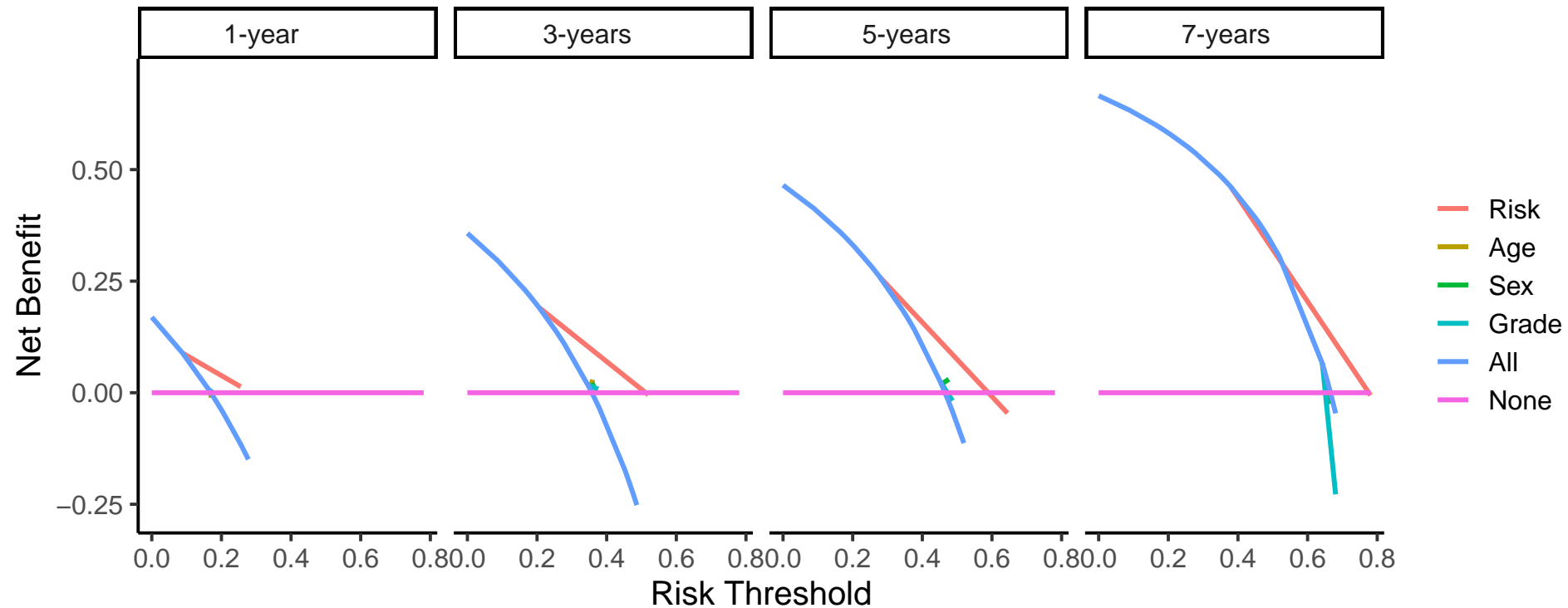

Supplement: Supplementary file 3 [file DataSheet1.ZIP › raw data/7-/DCA-train.pdf]
